# Electrostatically-sprayed carbon electrodes for high performance organic complementary circuits

**DOI:** 10.1038/s41598-022-19387-y

**Published:** 2022-10-07

**Authors:** Kazuyoshi Watanabe, Naoki Miura, Hiroaki Taguchi, Takeshi Komatsu, Hideyuki Nosaka, Toshihiro Okamoto, Shun Watanabe, Jun Takeya

**Affiliations:** 1 Department of Advanced Materials Science, Graduate School of Frontier Sciences, The University of Tokyo, 5-1-5 Kashiwanoha, Kashiwa, Chiba 277-8561 Japan; 2 NTT Device Technology Laboratories, Nippon Telegraph and Telephone (NTT) Corporation, 3-1 Morinosato-Wakamiya, Atsugi, Kanagawa 243-0198 Japan; 3 Material Innovation Research Center (MIRC), Graduate School of Frontier Sciences, The University of Tokyo, Kashiwa, Chiba 277-8561 Japan; 4 Precursory Research for Embryonic Science and Technology (PRESTO), Japan Science and Technology Agency (JST), Kawaguchi, Saitama 332-0012 Japan; 5 International Center for Materials Nanoarchitectonics (WPI-MANA), National Institute for Materials Science (NIMS), Tsukuba, 305-0044 Japan

**Keywords:** Electronic devices, Electrical and electronic engineering

## Abstract

Organic thin-film transistors (OTFTs) are promising building blocks of flexible printable electronic devices. Similar to inorganic FETs, OTFTs are heterostructures consisting of metals, insulators, and semiconductors, in which nanoscale interfaces between different components should be precisely engineered. However, OTFTs use noble metals, such as gold, as electrodes, which has been a bottleneck in terms of cost reduction and low environmental loading. In this study, we demonstrate that graphite-based carbon electrodes can be deposited and patterned directly onto an organic single-crystalline thin film via electrostatic spray coating. The present OTFTs exhibited reasonably high field-effect mobilities of up to 11 cm^2^ V^−1^ s^−1^ for p-type and 1.4 cm^2^ V^−1^ s^−1^ for n-type with no significant deterioration during electrostatic spray processes. We also demonstrate two significant milestones from the viewpoint of material science: a complementary circuit, an inverter consisting of p- and n-type OTFTs, and an operatable metal-free OTFT composed of fully carbon-based materials. These results constitute a key step forward in the further development of printed metal-free integrated circuits.

## Introduction

Thin-film transistors (TFTs) are one of the most important building blocks of electronic circuits^1–3^, where heterointerfaces between various components such as metals, semiconductors, and insulators play predominant roles in their performance^4–7^. TFT manufacturing processes require sequential deposition of these components, which is likely to hamper the reliable production of integrated devices. For TFTs with solution-processable organic semiconductors (OSCs), in particular, heterointerface engineering can be more deleterious because it should be compatible with printing technology^8,9^. With recent developments in chemistry^10–14^ and device engineering^15–20^ related to printed electronics, the performance of solution-processed OTFTs has been improving. In particular, for single-crystalline thin films consisting of a few monolayers of OSCs, reasonably high field-effect mobilities > 10 cm^2^ V^−1^ s^−1^ with excellent environmental stability have been achieved^15–17,21,22^. The improved manufacturing process allows for the production of large crystalline membranes with areal coverages of up to 100 cm^2^, which further facilitates the ideal production of reliable integrated circuits^16^.

Generally, OTFTs require sequential deposition of metal electrodes either on the top or on the periphery of the OSC thin films. Gold electrodes are often employed as the source, drain, and gate electrodes. There are various reasons for this: (1) the work function of gold (~ 5.0 eV) likely matches with the valence band edge (equivalent to the highest occupied molecular orbital (HOMO) of most p-type OSCs), (2) high-quality gold electrodes can be deposited by vacuum deposition, and (3) gold electrodes possess high environmental stability even though they are in the shape of ultrathin films. In particular, the quality of the gold/OSC interface is known to dominate the carrier injection properties and interfacial contact resistance^15,21^. Although electrodes based on solution-processed conductive polymers, such as PEDOT:PSS, have been studied previously^23^, there are limited studies on substitutes for gold electrodes, which is a bottleneck in terms of cost reduction and low environmental loading in printed, flexible electronics.

In this study, we demonstrate that graphite-based carbon can be deposited and patterned directly onto single-crystalline OSC thin films via electrostatic spray coating and works as an efficient contact electrode for both p- and n-type OTFTs. The OTFTs exhibit excellent transistor characteristics with high field-effect mobilities of up to 11 cm^2^ V^−1^ s^−1^ for p-type and 1.4 cm^2^ V^−1^ s^−1^ for n-type OTFTs, a near-zero turn-on voltage, negligible hysteresis, and an on–off current ratio of approximately 10^6^, which are comparable to those of gold-contact OTFTs^14,16,24^. In addition, a complementary inverter consisting of p- and n-type OTFTs was successfully operated at a supplied voltage (*V*_dd_) of 5–15 V, which is one of the first organic complementary circuits to be operated with graphite-based carbon electrodes. We also operated a metal-free OTFT comprising only carbon-based materials, such as OSC, carbon contact/gate electrodes, organic polymer insulators, and organic polymer substrates. The results will be the basis for the further development of printed, metal-free, complementary integrated circuits.

## Results

### Fabrication of OTFTs with carbon contact electrodes

**Figure 1:**
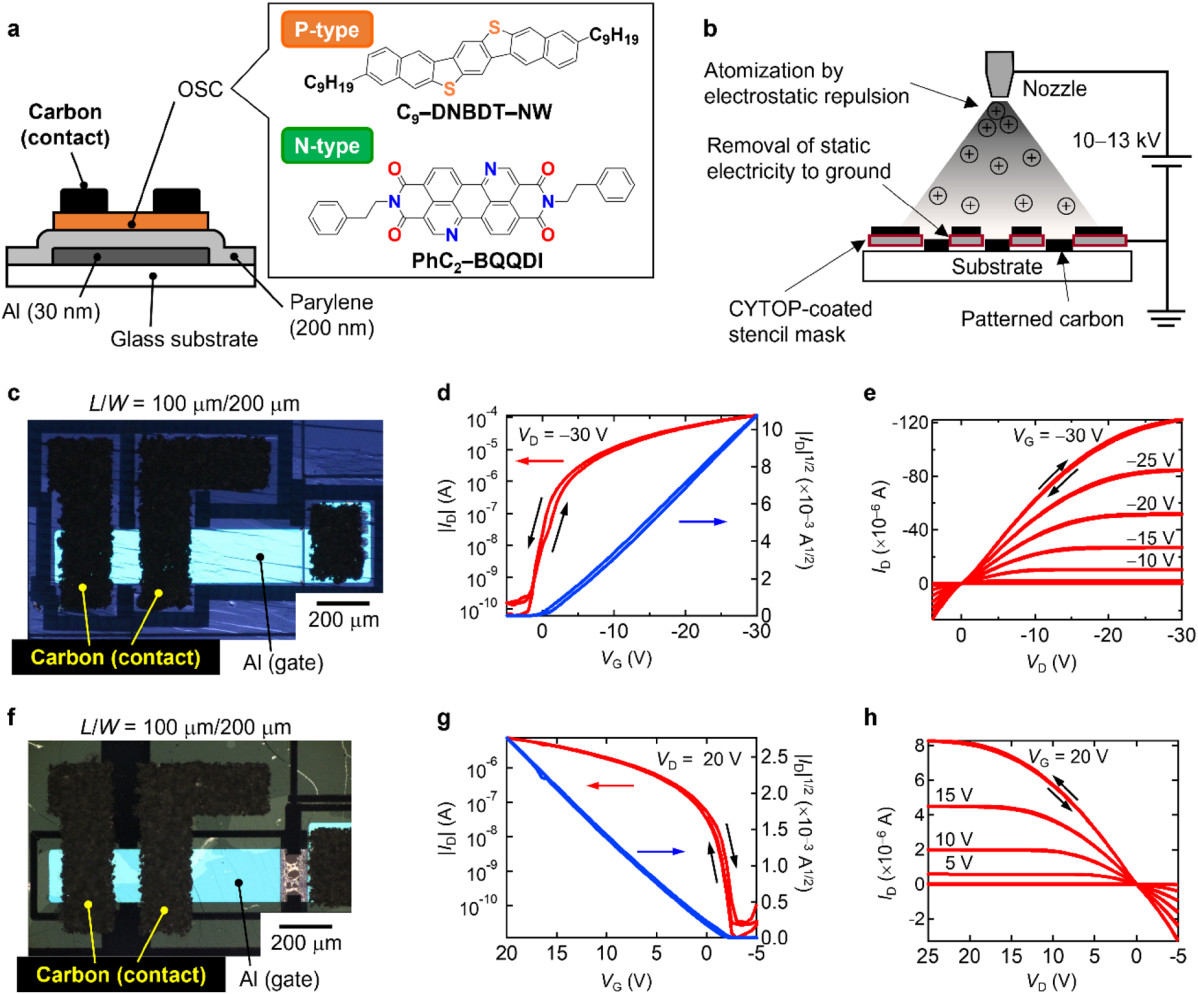
Configuration and transistor characteristics of p- and n-type OTFTs with carbon contact electrodes. (**a**) Device configuration of OTFTs with carbon contact electrodes, in which a single-crystalline thin film of either p-type C_9_–DNBDT–NW or n-type PhC_2_–BQQDI was employed as the OSC layer. (**b**) Schematics of electrostatic spray coating of a carbon suspension including graphite powder and carbon black. The carbon suspension was atomized by electrostatic repulsion due to the charging nozzle to which a high voltage of 10–13 kV was applied. The carbon was patterned on a target substrate through a CYTOP-coated stencil mask. (**c**) Transfer characteristics in the saturation regime (*V*_D_ = − 30 V) and (**d**) output characteristics of a p-type OTFT including C_9_–DNBDT–NW as the OSC layer and carbon as the contact electrodes. (**e**) Polarized optical microscopy (POM) image of the p-type OTFT under cross-Nicol condition. (**f**) Transfer characteristics in the saturation regime (*V*_D_ = 20 V) and (**g**) output characteristics of the n-type OTFT with PhC_2_–BQQDI as the OSC layer and carbon as the contact electrodes. (**h**) POM image of the n-type OTFT under cross-Nicol condition. The channel length (*L*) and width (*W*) of both OTFTs were 100 μm and 200 μm, respectively.

We employed our benchmarked small-molecule OSCs, 3,11-dinonyldinaphtho[2,3-*d*:2’,3’-*d*’]benzo[1,2-*b*:4,5-*b*’]dithiophene (C_9_–DNBDT–NW)^25^ and *N*,*N’*-diphenethyl-3,4,9,10-benzo[*de*]isoquinolino[1,8-*gh*]quinolinetetracarboxylic diimide (PhC_2_–BQQDI)^14^ for the p- and n-type OTFTs, respectively. Figure 1a shows the device configuration of the bottom-gate top-contact OTFTs fabricated using the above OSCs and carbon contact electrodes. Al (*t* = 30 nm) and parylene (*t* = 200 nm) were sequentially deposited on Eagle XG glass as the gate electrode and insulator, respectively. The capacitance per unit area (*C*_i_) was evaluated as 13.7 nF cm^−2^ based on its thickness and relative permittivity *ε*_r_ = 3.1. Single-crystalline OSC thin films, fabricated by continuous edge-casting^26^, were transferred onto the top and then patterned by laser etching. The fabrication procedure is described in the “Materials and methods” section.

The goal of this study was successful deposition and patterning of carbon contact electrodes onto OSC thin films without any deterioration in the quality of the OSC single crystal. To achieve this, we adopted an electrostatic spray coating of a carbon suspension, Dotite XC-9089, which is a ternary mixture; graphite as the main electric conductor; carbon black as the conductive additive; and a polyacrylate binder in butyl acetate. Butyl acetate was selected as a damage-free solvent because of its good wettability and poor solubility in both C_9_–DNBDT–NW and PhC_2_–BQQDI single crystals. An electrostatic spray (Fig. 1b), in which a high voltage of 10–13 kV is applied to a spray nozzle to atomize the ejection by electrostatic repulsion, can efficiently change the carbon suspension into a mist, resulting in quick evaporation of the solvent. This also contributes to reducing damage to the OSC films. Through electrostatic spray coating, the carbon electrode was patterned on a substrate of up to 100-mm × 100-mm using a stencil mask coated with a solvophobic CYTOP polymer, which prevented the carbon suspension from spreading out under the mask. According to the patterning method, a graphite-based carbon contact electrode was successfully formed on both the laser-etched OSC films, as shown in the polarized optical microscopy (POM) images of the resulting p- and n-type OTFTs in Fig. 1c,f. The channel length (*L*) and width (*W*) were 100 μm and 200 μm, respectively, so that *L*/*W* was 0.5 for both of the OTFTs. The patterning method also successfully reduced the OTFT channel length to 50 μm.

### Evaluation of OTFTs with carbon contact electrodes

The transistor characteristics of the p- and n-type OTFTs are shown in Fig. 1d,e,g,h. The effective field-effect mobilities (*μ*_eff_) extracted from the transfer curves in the saturation regime were 10.9 cm^2^ V^−1^ s^−1^ (9.8 ± 0.6 cm^2^ V^−1^ s^−1^, *N* = 6) and 1.4 cm^2^ V^−1^ s^−1^ (1.4 ± 0.2 cm^2^ V^−1^ s^−1^, *N* = 3) for the p- and n-type OTFTs, respectively. These values are as high as those previously reported for OTFTs consisting of commonly-used gold contact electrodes and the same OSCs^14,16,24^. The threshold and turn-on voltages (*V*_th_ and *V*_on_) were estimated as − 2.3 and + 1.5 V for the p-type OTFT, and − 0.2 and − 2.0 V for the n-type OTFT, respectively, indicating that both OTFTs turned on at a voltage of almost zero. In addition, the transfer curves in the saturation regime and the output curves exhibited negligible hysteresis and a high on–off current ratio of more than 10^6^, which is textbook-like behavior. Therefore, it should be emphasized that electrostatically deposited carbon electrodes are excellent substitutes for conventional noble metal contact electrodes in OTFTs. This is also supported by the photoelectron yield spectroscopy (PYS) results in Supplementary Fig. 1 online, which revealed that the carbon suspension exhibits a high work function *Φ*_C_ = 5.28 eV, which is as high as that of gold^27^. Furthermore, these results imply that the electrostatic spray coating of OSC thin films is a damage-free process even though the carbon suspension is directly sprayed onto surface of the films, resulting in a functional heterostructure between the carbon electrode and the OSCs.

In this report, we intentionally designed OFETs with the relatively large *L* (on the range of 100 μm). It is predominantly because of the restrictions of stencil mask. We found that the actual channel length on the substrate is slightly larger (approximately 5 μm) than the designed channel length, which corresponds to the length on the stencil mask. This clearly indicates that the pattern edges extend by deposition of carbon particles shaded from the mask pattern, i.e., the shadow effect. It is feasible to improve the patterning accuracy using a photolithography process.

### Complementary inverter with carbon contact electrode

**Figure 2:**
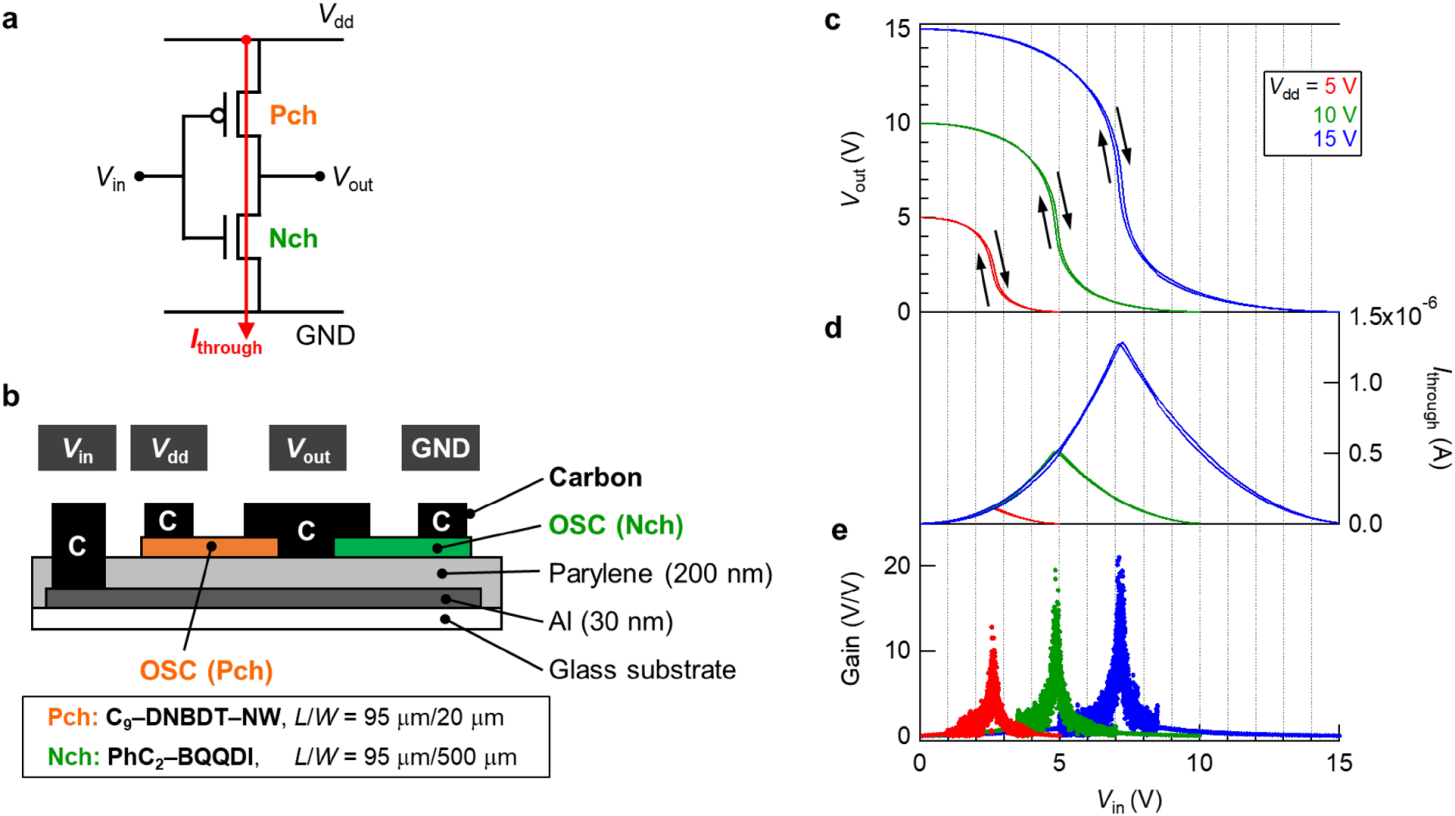
Complementary inverter with carbon contact electrodes. (**a**) Circuit diagram and (**b**) device configuration of a complementary inverter consisting of one p-type and one n-type OTFT with carbon contact electrodes. (**c**) Voltage transfer curves, (**d**) shoot-through current, and (**e**) voltage gain in the *V*_dd_ range of 5–15 V.

A complementary inverter consists of one p-type and one n-type OFET; hence, it is regarded as the simplest complementary circuit. Thus, the operation of complementary inverters based on these OTFTs, in which one p-type and one n-type OTFT were connected to each other, as depicted in Fig. 2a,b, was demonstrated. Figure 2c shows the voltage transfer curves obtained at supply voltages (*V*_dd_) of 5, 10, and 15 V. Full rail-to-rail swing, small hysteresis, and on–off switching behavior were observed at all *V*_dd_ values owing to the balanced OTFTs in the complementary inverter. The switching voltage, corresponding to the voltage when *V*_out_ = *V*_in_ (*V*_out_: output voltage, and *V*_in_: input voltage), was almost half the value of the applied *V*_dd_; for example, the switching voltage was 4.89 V at *V*_dd_ of 10 V. The maximum signal gain (*Gain* = ∂*V*_out_/∂*V*_in_) reached 20 at *V*_dd_ of 10 V when *V*_in_ was around the switching voltage (Fig. 2e). In addition, the shoot-through current (*I*_through_) is plotted as a function of *V*_in_ in Fig. 2d. At a 10 V operation, *I*_through_ at *V*_in_ = 0 V and *V*_in_ = *V*_dd_ = 10 V was approximately 2 nA, resulting in a minimum static power consumption of 20 nW. Furthermore, the *I*_through_ exhibited a maximum value of 0.52 μA at the switching voltage. As a result, the simplest complementary circuit, the inverter, was successfully operated using a carbon contact electrode. All properties are summarized in Table 1.

**Table 1:** Characteristics of the complementary inverter with carbon contact electrodes.

*V*_dd_ (V)	*V*_sw_ (V)	*I*_through_ (A)	*Gain*_max_ (V/V)	*I*_peak_ × *V*_dd_ (W)
15	7.19	1.28 × 10^−6^	21.0	1.93 × 10^−5^
10	4.89	5.19 × 10^−7^	19.5	5.19 × 10^−6^
5	2.64	1.11 × 10^−7^	12.8	5.56 × 10^−7^

### Metal-free OTFTs with carbon contact and gate electrodes

**Figure 3:**
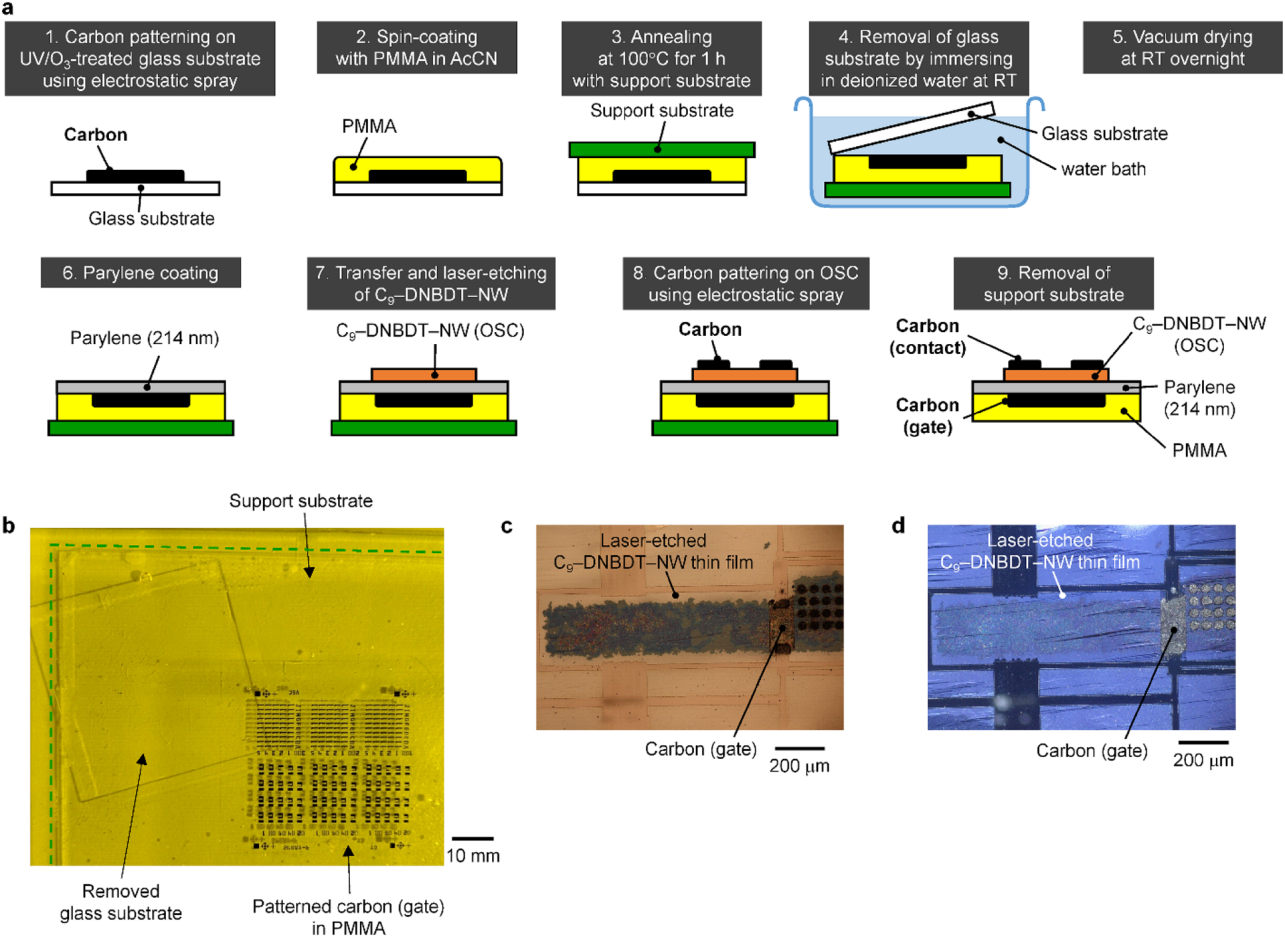
Fabrication of metal-free OTFTs. (**a**) Sequential fabrication procedure of a metal-free OTFT composed of C_9_–DNBDT–NW as the OSC, carbon as the contact and gate electrodes, parylene as the hydrocarbon polymeric insulator, and PMMA as the substrate. (**b**) Photo of a patterned carbon/PMMA film being removed from a glass substrate in a water bath. (**c**, **d**) POM images of a C_9_–DNBDT–NW thin film transferred onto a parylene/carbon/PMMA film and then laser-etched. Observed under (**c**) open-Nicol and (**d**) cross-Nicol conditions, respectively.

We also demonstrated metal-free OTFTs by replacing the aforementioned parylene/Al/glass substrate with a fully carbon-based parylene/XC-9089/poly(methyl methacrylate) (PMMA). The sequential fabrication procedure is illustrated in Fig. 3a. The carbon gate electrode was patterned on a UV/O_3_-treated Eagle XG glass substrate by electrostatic spray coating of a carbon suspension XC-9089 as described above. The carbon electrode was spin-coated with a 20 wt% solution of PMMA (*M*_w_ = 120,000) in acetonitrile and then baked on a hot plate at 80 °C for 30 min. Spin-coating was performed twice to obtain a thick, self-standing PMMA film. A support substrate composed of poly(dimethylsiloxane) (PDMS) was placed on top of the PMMA film, followed by annealing at 100 °C for 1 h. The entire substrate was turned upside down and immersed in deionized water at RT, resulting in the removal of the UV/O_3_-treated glass substrate (Fig. 3b). After vacuum drying overnight at RT, a carbon gate electrode embedded in a PMMA film was obtained. The arithmetic average surface roughness (*R*_a_) of the carbon gate electrode was evaluated as 30–60 nm with imaging interferometric microscopy, resulting in a relatively smooth surface regardless of the average graphite particle size of 3 μm. This is because the Eagle XG glass acted as a smooth surface template, and both the carbon black and the polymeric binder filled the gaps among the graphite particles. The following processes, such as parylene coating, transfer of the C_9_–DNBDT–NW thin film, and laser etching, were performed using the same procedure as above. As shown in the POM images obtained under open-Nicol (Fig. 3c) and cross-Nicol conditions (Fig. 3d), the OSC thin film was transferred above the carbon gate without experiencing serious damage, such as channel-crossing cracks. The *C*_i_ of the gate insulator, parylene, with a thickness of 214 nm, was evaluated as 12.8 nF cm^−2^. Finally, integrated OTFTs on a self-standing PMMA film were obtained by forming a carbon contact electrode by means of electrostatic spray coating and then removing the PDMS support substrate. It is noteworthy that all the components, namely the substrate, electrodes, gate insulator, and semiconductor, are carbon-based materials rather than metals.

**Figure 4:**
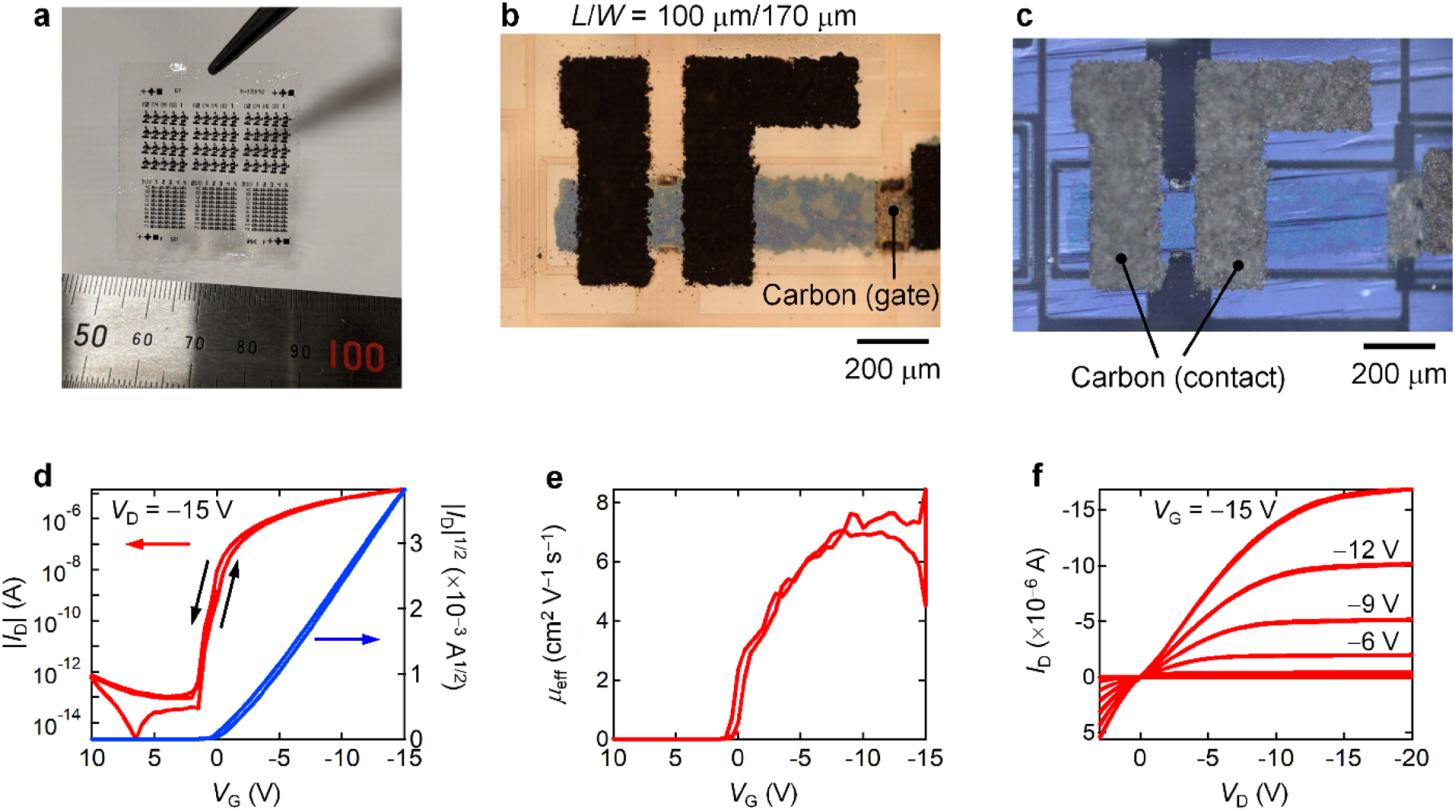
Characteristics of metal-free OTFTs. (**a**) Photo of metal-free OTFTs on a 30-mm by 30-mm self-standing PMMA film. (**b**, **c**) POM images of the metal-free OTFT under (**b**) open-Nicol and (**c**) cross-Nicol conditions, respectively. (**d**) Transfer curve in the saturation regime (*V*_D_ = − 15 V), (**e**) corresponding effective mobilities plotted as functions of *V*_G_, and (**f**) output curve of the metal-free OTFT.

Figure 4a shows a 30-mm × 30-mm self-standing PMMA film, which is colorless and transparent, except for the carbon electrode moieties. Figure 4b,c show POM images of a metal-free p-type OTFT on the PMMA film observed under open-Nicol and cross-Nicol conditions, respectively. *L*/*W* of the channel was 100 μm/170 μm. The transistor characteristics of the metal-free OTFT were also investigated. Figure 4d–f show the transfer curves in the saturation regime, the corresponding *μ*_eff_, and the output curves, respectively. The characteristics were a slight improvement on those of the aforementioned Al-gate p-type OTFT; for example, *V*_th_ and *V*_on_ were estimated to be − 1.5 and + 1.0 V, indicating that the turn-on voltage was close to zero. Furthermore, the metal-free OTFT exhibited a high on–off current ratio of more than 10^8^ and a relatively high *μ*_eff_ of 7.3 cm^2^ V^−1^ s^−1^ (4.4 ± 2.1 cm^2^ V^−1^ s^−1^, *N* = 11). Although this metal-free fabrication process can be universally applicable both to p-type and to n-type OSCs, the quality of single crystalline thin films of n-type OSCs has room for improvement, which clearly causes the lack of reproducibility in manufacturing CMOS circuits. Overall, these results suggest that metal-free, fully carbon-based OTFTs can be realized.

## Discussion

In this study, we developed an electrostatic spray coating method to pattern graphite-based carbon contact electrodes onto p- and n-type single-crystalline OSC thin films without serious damage. The resultant p- and n-type OTFTs exhibited excellent transistor characteristics represented by high effective field-effect mobilities of 11 cm^2^ V^−1^ s^−1^ for p-type OTFTs and 1.4 cm^2^ V^−1^ s^−1^ for n-type OTFTs, respectively. These characteristics are comparable to those of common OTFTs possessing gold contact electrodes, strongly indicating that cheap, widely distributed, and easily accessible carbon can replace precious metals such as gold, silver, and platinum as effective contact electrode materials for OTFTs. The present carbon suspension can be used for other printing techniques, such as inkjet printing, and offset printing. Currently, we employed the commercially available graphite powder with the average particle size of 3 μm. Further reduction in the particle size allows a finer patterning, which will be a key issue for the future. Moreover, we successfully operated the simplest complementary circuit, an inverter, consisting of the p- and n-type OTFTs with supply voltages of 5–15 V. The spray-coated area is easily enlarged by extending the nozzle scanning distance, and we have already succeeded in patterning the carbon electrode on a 50-mm × 50-mm substrate. Herein, we also fabricated and operated a fully carbon-based OTFT composed of a p-type OSC, carbon electrodes, hydrocarbon polymeric insulators, and a PMMA substrate. Hence, the fabrication of metal-free integrated circuits using carbon electrodes will be realized in the near future, and OTFT applications in flexible printable electronic devices will achieve further progress.

## Materials and methods

### Materials

The p-type OSC, C_9_–DNBDT–NW, was synthesized and purified in-house. The n-type OSC and PhC_2_-BQQDI were purchased from FUJIFILM Wako Pure Chemical Corporation. The carbon suspension Dotite XC-9089 (Fujikura Kasei Co., Ltd.) was prepared by mixing graphite powder (average particle size: 3 μm) and carbon black with polyacrylate binder in butyl acetate. The solid content was approximately 20 wt%, with a weight ratio of graphite:carbon black:binder of 3:1:1. All the other chemicals and materials used were commercially available.

### Carbon patterning with electrostatic spray coating

Electrostatic spraying was performed using a Micro Mist Coater PDR-06 (Nagase Techno-Engineering Co. Ltd.). Both sides of a stainless steel stencil mask were blade-coated with a fluorinated polymer, CYTOP (AGC Inc.), to make the surface of the mask solvophobic. After being washed with 1,1,1,2,2,3,3,4,4,5,5,6,6-tridecafluorooctane to remove excess CYTOP, the mask was placed on the target substrate and connected to the ground. The masked substrate was heated to 80 °C and then electrostatically spray-coated 10 times with XC-9089 by supplying the carbon suspension at a flow rate of 0.10 mL/min into a spray nozzle to which a 10–13 kV voltage had been applied. The nozzle scan speed was 100 mm/s. After it was vacuum dried at 70 °C for 1 h, the patterned carbon on the target substrate was obtained by removing the stencil mask.

### Fabrication of OTFTs and complementary inverter possessing carbon contact electrode

p- and n-type OTFTs with carbon contact electrodes were fabricated on Eagle XG glass (Corning Inc.) substrates with a thickness of 0.7 mm. On a glass substrate cleaned by O_2_ plasma, 30-nm-thick Al was deposited and patterned by e-beam evaporation through a stainless stencil mask (*t* = 50 μm). The Al layer was encapsulated by a 200-nm-thick parylene diX-SR (KISCO Ltd.), serving as a gate insulator. As described in our previous study^26^, single-crystalline thin films of p- and n-type OSCs were obtained by continuous edge-casting of a 0.02 wt% solution of C_9_–DNBDT–NW in 3-chlorothiophene on UV/O_3_-treated Eagle XG glass at 90 °C, and a 0.02 wt% solution of PhC_2_–BQQDI in 1-chloronaphthalene on nano-ground glass^24^ at 148 °C, respectively. After edge casting, each substrate was cut into pieces. The C_9_–DNBDT–NW film was placed directly face-down on the parylene/Al/glass substrate and then transferred to the substrate by applying a few drops of ultra-pure water between the two substrates^22^. Meanwhile, the PhC_2_–BQQDI film was transferred onto the parylene/Al/glass substrate via a relay substrate made of PDMS to avoid serious damage being made to the film^24^. After being vacuum-dried at 80 °C for 10 h, both of the transferred OSC films were patterned by a laser ablation process using an yttrium–aluminum–garnet (YAG) laser and a UV picosecond laser (*λ* = 355 nm). The channel length (*L*) and width (*W*) were 100 and 200 μm, respectively, and *L*/*W* was 0.5. The carbon contact electrodes were patterned onto the OSC films by electrostatic spray coating through a CYTOP-coated stainless steel stencil mask, as described above.

Complementary inverters with carbon contact electrodes were fabricated using the same procedure as above, but *L*/*W* was 95 μm/20 μm for p-type C_9_–DNBDT–NW and 95 μm/500 μm for n-type PhC_2_–BQQDI.

### Electrical measurements

All the electrical measurements were performed using a semiconductor characterization system, 4200-SCS (Keithley), under dark and ambient conditions. The effective field-effect mobility, *μ*_eff_, in the saturation regime was determined from the transfer characteristics using

$${I}_{{\mathrm{D}}, {\mathrm{sat}}}= \frac{{\mu }_{{\mathrm{eff}}}W{C}_{i}}{2L}{({V}_{{\mathrm{G}}}-{V}_{{\mathrm{th}}})}^{2}$$

where *I*_D,sat_, *L*, *W*, *C*_i_, *V*_G_, *V*_th_, and *V*_D_ are the drain current in the saturation regime, channel length, channel width, capacitance per unit area, gate voltage, threshold voltage, and drain voltage, respectively. The values of *C*_i_ were determined from the thickness and relative permittivity of the gate insulator parylene diX-SR.

## Supplementary Information

Supplementary Figure 1.

## Data Availability

The data that support the plots in this paper and other findings of this study are available from the corresponding author (Kazuyoshi Watanabe; kaz-watanabe@edu.k.u-tokyo.ac.jp) upon request.
