# Supplementary material for: Electrostatically-sprayed carbon electrodes for high performance organic complementary circuits
Source: Sci Rep. 2022 Oct 7;12:16009. doi: 10.1038/s41598-022-19387-y (PMC9546853; doi:10.1038/s41598-022-19387-y)
Supplement: Supplementary file 1 — Supplementary Figure 1. [file 41598_2022_19387_MOESM1_ESM.pdf]

Supplementary information

Electrostatically-sprayed carbon electrodes for high performance organic complementary circuits

Kazuyoshi Watanabe[1]*, Naoki Miura[2], Hiroaki Taguchi[2], Takeshi Komatsu[2], Hideyuki Nosaka[2], Toshihiro Okamoto[1,3,4], Shun Watanabe[1,3]* & Jun Takeya[1,3,5]*

[1] Department of Advanced Materials Science, Graduate School of Frontier Sciences, The University of Tokyo, 5-1-5 Kashiwanoha, Kashiwa, Chiba, 277-8561, Japan.
[2] NTT Device Technology Laboratories, Nippon Telegraph and Telephone (NTT) Corporation, 3-1 Morinosato-Wakamiya, Atsugi, Kanagawa, 243-0198, Japan.
[3] Material Innovation Research Center (MIRC), Graduate School of Frontier Sciences, The University of Tokyo, Kashiwa, Chiba 277-8561, Japan
[4] Precursory Research For Embryonic Science and Technology (PRESTO), Japan Science and Technology Agency (JST), Kawaguchi, Saitama 332-0012, Japan
[5] International Center for Materials Nanoarchitectonics (WPI-MANA), National Institute for Materials Science (NIMS), Tsukuba 305-0044, Japan

* Corresponding authors: Kazuyoshi Watanabe (kaz-watanabe@edu.k.u-tokyo.ac.jp); Shun Watanabe (swatanabe@edu.k.u-tokyo.ac.jp); Jun Takeya (takeya@k.u-tokyo.ac.jp).

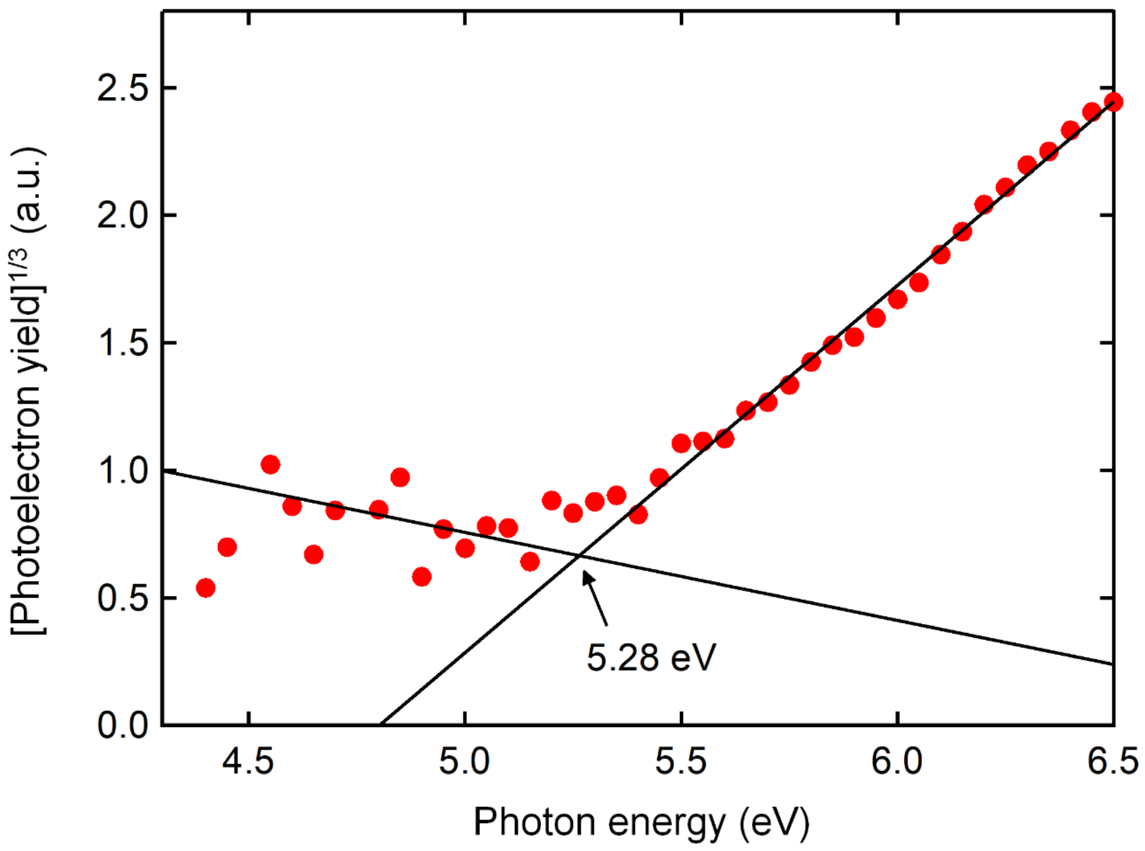

Supplementary Figure 1. Ionization potential of graphite-based carbon resulting from photoelectron yield spectroscopy (PYS). The carbon suspension XC-9089 was electrostatically sprayed on a glass substrate and then dried at 80°C for 30 min to obtain film-like carbon sample. The PYS measurement was carried out under vacuum.
